# Smoking-induced gut microbial dysbiosis mediates cancer progression through modulation of anti-tumor immune response

**DOI:** 10.1016/j.isci.2025.112002

**Published:** 2025-02-11

**Authors:** Prateek Sharma, Tejeshwar Jain, Ali Sorgen, Srikanth Iyer, Mohammad Tarique, Pooja Roy, Saba Kurtom, Vrishketan Sethi, Ejas P. Bava, A.K. Gutierrez-Garcia, Utpreksha Vaish, Dhanisha Sulekha Suresh, Preeti Sahay, Dujon Edwards, Jumana Afghani, Satwikreddy Putluri, Karthik Reddy Kami Reddy, Chandra Sekhar Amara, Abu Hena Mustafa Kamal, Anthony Fodor, Vikas Dudeja

**Affiliations:** 1 Department of Surgery, University of Alabama, Birmingham, AL 35233, USA; 2 Department of Bioinformatics and Genomics, University of North Carolina at Charlotte, Charlotte, NC 28223, USA; 3 Sylvester Cancer Center, Department of Surgery, University of Miami, Miami, FL 33136, USA; 4 Department of Molecular and Cellular Biology, Baylor College of Medicine, Houston, TX 77030, USA

**Keywords:** Biological sciences, Immunology, Microbiome, Cancer, Metabolomics

## Abstract

Cigarette smoke exposure (CSE) increases the risk for a plethora of cancers. Recent evidence indicates that the gut microbiome can influence cancer progression by immune system modulation. Since CSE alters the gut microbiome, we hypothesized that the gut microbiome serves as a causative link between smoking and cancer growth. Through a combination of syngeneic animal models and fecal microbiota transplantation studies, we established an essential role for smoke-induced dysbiosis in cancer growth. 16s rRNA sequencing and liquid chromatography-mass spectrometry indicated a unique CSE-associated microbial and metabolomic signature. Immunophenotyping of tumor specimens and experiments in Rag1-KO and CD8-KO demonstrated that smoke-induced tumor growth requires functional adaptive immunity. Finally, utilizing gut microbial ablation strategies with broad- and narrow-spectrum antibiotics, we demonstrated the reversal of phenotypic effects of CSE. Our study provides evidence for gut microbiome as an actionable target to mitigate CSE-induced tumor promotion.

## Highlights

• Cigarette smoke exposure (CSE) induces unique gut microbial and metabolomic signatures
• Gut microbial dysbiosis induced by CSE is essential for its tumor-promoting effects
• Fecal microbiome transfer from CSE mice into smoke-naïve mice drives increased tumor growth
• Targeting the dysbiotic gut microbiome ameliorates tumor-promoting effects of CSE

## Graphical abstract

**Figure:**
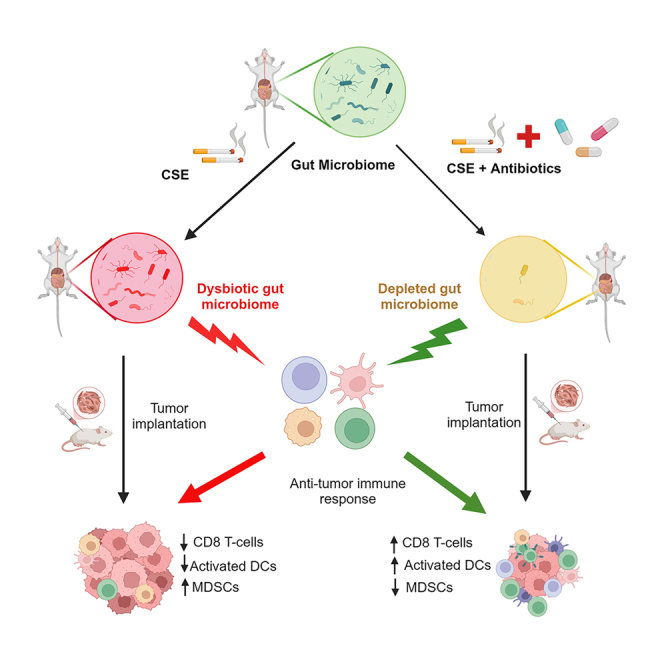

## Introduction

Cigarette smoke exposure (CSE) has been extensively investigated as a risk factor for cancer development.^1^^,^^2^ Potential carcinogens detected in cigarette smoke^3^ have been postulated to aid cancer initiation and progression through genotoxic effects and reactive oxygen species generation.^4^^,^^5^ Recent studies have also explored the direct effects on cell proliferation through binding to nicotinic acetylcholine receptors (nAChRs).^6^ However, the molecular mechanisms underlying the tumor-promoting effects of CSE remain elusive, and there is a dearth of clinically actionable targets.

We have witnessed the recent emergence of the gut microbiome as a key regulator of human physiology, and its perturbations linked to ailments such as gastrointestinal diseases,^7^ neurological diseases,^8^ behavioral conditions,^9^ and malignancies.^10^^,^^11^^,^^12^^,^^13^^,^^14^^,^^15^ Recent research has demonstrated that smokers, too, harbor a dysbiotic microbiome.^16^^,^^17^^,^^18^ However, the implications of this changed gut microbiota have not yet been explored. We, along with others, have shown that the gut microbiome significantly drives cancer progression by affecting the anti-tumor immune response.^14^^,^^19^ Targeting the microbiome as a strategy to enhance the efficacy of immunotherapy in multiple cancers is being explored.^20^ In light of these findings, we hypothesized that the gut microbiome might be the missing link between smoking and cancer progression. Herein, we elucidate a novel mechanism for smoking-dependent cancer progression via the gut microbiome through modulation of the adaptive immune system. We characterize the unique microbiome and metabolome potentiating this smoking-dependent cancer progression and present an actionable target to mitigate the harmful effects of cigarette smoke.

## Results

### The gut microbiome is required and sufficient to mediate cigarette-smoke-induced cancer progression

**Figure 1:**
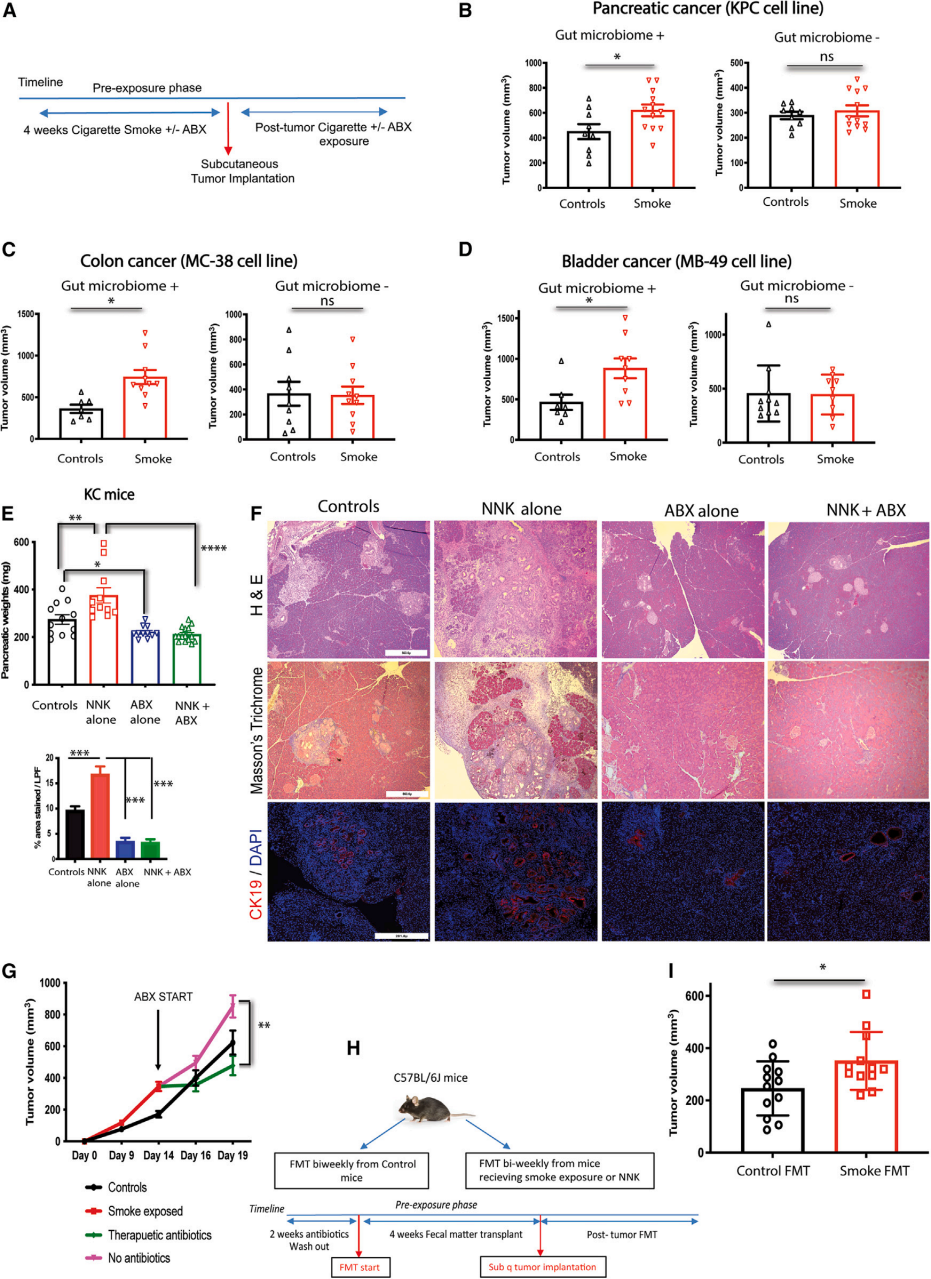
The gut microbiome is required and sufficient to promote cigarette-smoke-induced cancer progression (Also refer to Figure S1) (A) Schematic timeline of experimental design of the subcutaneous tumor model in C57BL/6J mice showing the pre-exposure phase of 4 weeks before tumor implantation followed by continued exposure of CSE with or without gut microbiome depletion. (B–D) Subcutaneous tumor volumes at the endpoint in mice exposed to CSE with or without gut microbiome depletion using (B) pancreatic cancer (data representative of three independent experiments), (C) colon cancer, and (D) bladder cancer cell lines, respectively (*n* = 8–12 per group). (Left panels (B–D): gut microbiome present; right panels (B–D): gut microbiome depleted state). (E) Pancreatic weights of *LSL-Kras*^*G12D/+*^*;Pdx-1-Cre* (KC mice) at 4 months of age after exposure with NNK for 2 months with or without gut microbiome depletion. (*n* = 11–12 per group). (F) (Top panel) Representative H&E stain of KC mice pancreas showing significant ductal proliferation and fibrosis in the pancreas of mice exposed to NNK compared to controls, gut microbiome depleted, and gut microbiome depleted NNK-exposed mice (scale bar, 563.6 μm); (Middle panel) Trichrome staining showing fibrosis in the pancreas of KC mice. Quantification (shown on the left) done as described in methodology (*n* = 4 mice per group; scale bar, 563.6 μm); (bottom panel) cytokeratin-19 staining showing ductal proliferation among various groups. (*n* = 4 per group; scale bar, 281.8 μm). (G) Tumor kinetics of control and CSE mice with CSE mice implanted with Mc-38 cell line randomized at day 14 to receive no antibiotics vs. broad-spectrum antibiotics. (*n* = 10 controls: *n* = 20 smoke-exposed before randomization at day 14). (H) Schematic timeline of FMT studies in C57BL/6J mice randomized to receive FMT from control mice or CSE/NNK-exposed mice. (I) Subcutaneous tumor volumes at the endpoint in recipient mice getting FMT from control donors vs. CSE donors (*n* = 12 per group). Data representative of two independent experiments. CSE: cigarette smoke exposure; NNK: 4-(methylnitrosamino)-1-(3-pyridyl)-1-butanone; FMT: fecal microbiome transplant. ∗ *p* value <0.05; ∗∗*p* value <0.01; ∗∗∗ *p* value <0.001. Unpaired t test or ANOVA used as appropriate for statistical comparison. Data presented as mean ± SEM.

We first evaluated the effect of gut microbiome depletion on cigarette smoke exposure (CSE)-induced tumor progression. CSE was given to cancer-naive C57BL/6J wild-type (WT) mice with and without a broad-spectrum antibiotic cocktail to deplete the gut microbiome followed by subcutaneous cancer cell implantation (Figure 1A). CSE promoted tumor growth in KPC (pancreatic cancer), MC-38 (colon cancer), and MB-49 (bladder cancer) models in the presence of gut microbiome (Figures 1B–1D, left panels; Figure S1A). Interestingly, in gut-microbiome-depleted states, CSE failed to promote tumor growth (Figures 1B–1D, right panels). In another set of experiments, when the smoke analog nicotine-derived nitrosamine ketone (NNK; *N*-methyl-*N*-(4-oxo-4-pyridin-3-ylbutyl)nitrous amide), one of the most potent carcinogens in cigarette smoke,^21^ was administered intraperitoneally, gut microbiome depletion was still able to nullify any tumor-promoting effects across multiple cancer models (Figures S1B–S1D).

Next, we utilized genetic models with a predisposition to cancer formation to test our hypothesis (Figure S1E). NNK exposure to KC (*LSL-Kras*^*G12D/+*^*;Pdx-1-Cre*) mice, which are predisposed to spontaneous pancreatic intraepithelial neoplasia (PanIN) and pancreatic cancer development, resulted in increased mean pancreatic weights compared to mice receiving simultaneous NNK and broad-spectrum antibiotic treatment (Figure 1E). Histology showed significant fibrosis and increased CK-19 expression in the NNK-exposed group relative to the control group; however, simultaneous treatment with antibiotics to NNK-exposed mice significantly reduced fibrosis and ductal proliferation (Figure 1F). Similarly, A/J mice, which are highly susceptible to forming carcinogen-induced lung tumors, developed significantly more tumor nodules in the lungs of NNK-exposed mice with intact gut microbiomes when compared to those with depleted gut microbiomes (Figure S1F). To confirm whether this effect could be replicated in the setting of well-established tumors, we employed a therapeutic model of gut microbiome depletion where subcutaneous tumors were allowed to grow for 14 days under the influence of CSE before antibiotic depletion was initiated. The broad-spectrum antibiotic cocktail was able to significantly retard tumor growth in mice receiving CSE after only 5 days of treatment (Figure 1G).

To further evaluate if smoking-induced gut microbial dysbiosis is sufficient to cause smoke-induced tumor progression, we performed fecal microbiota transplants (FMTs) from tumor-naive control (smoke-free) WT donor mice and cigarette-smoke-exposed WT donor mice into recipient WT mice with depleted gut microbiome (Figure 1H). These recipient mice with reconstituted gut microbiomes were then challenged with subcutaneous KPC pancreatic cancer tumors. Mice receiving FMT from smoke-exposed mice had significantly higher mean tumor volumes than mice receiving FMT from control mice (Figure 1I). To further validate this finding, FMT was performed with NNK-exposed mice instead of CSE mice with similar results (Figure S1G). We measured serum cotinine concentrations (a byproduct of nicotine metabolism, which has been shown to correlate with smoke exposure^22^) in the donor mice as well as recipient mice post-FMT to ensure that smoke metabolites were not being passively transferred through the FMT. Serum cotinine levels were significantly elevated only in the CSE-exposed donor mice (25.8 ng/mL), consistent with the levels reported in the literature for mice undergoing smoke exposure.^5^ Mice receiving FMT from smoke donors had relatively undetectable levels (<0.1 ng/mL), making the transmission of nicotine through donor stool unlikely (Figure S1H). In addition, antibiotics also did not affect serum cotinine levels (Figure S1I). A recent report has indicated that gut microbes can metabolize and degrade nicotine.^23^ To verify whether antibiotics treatment was affecting nicotine levels in the gut, we analyzed the nicotine levels in ileal contents. Although mice exposed to cigarette smoke had higher levels of nicotine in the ileal contents, these remained unchanged in mice where gut microbiome was sterilized with antibiotics, indicating that nicotine metabolism was not affected by gut microbiome in our model (Figure S1J). These findings indicate that the gut microbiome is required and sufficient to induce smoking-dependent tumor progression.

### Modulation of adaptive and innate immune response drives the smoking-induced-dysbiosis-mediated cancer progression

**Figure 2:**
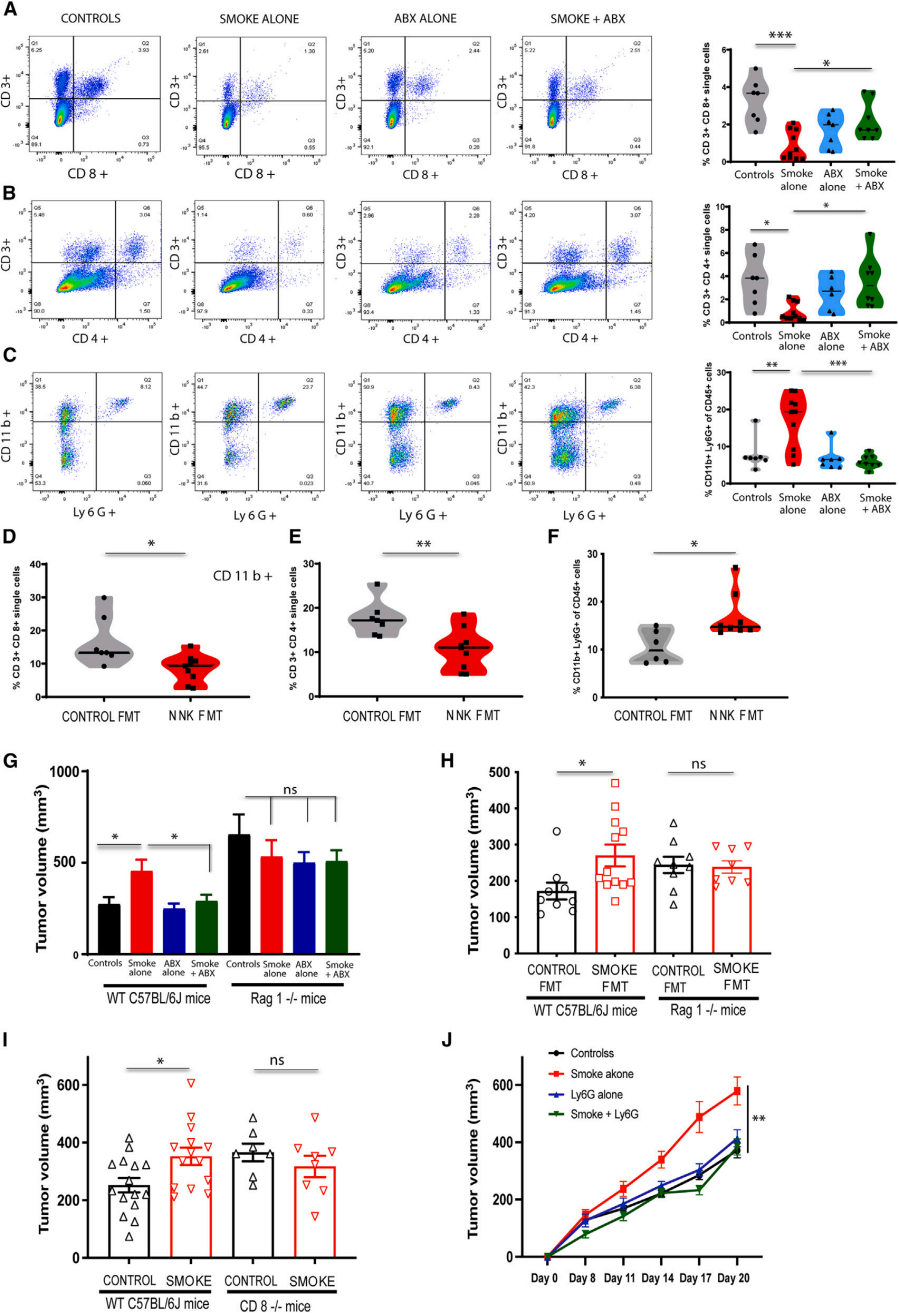
Modulation of adaptive immune response drives the smoking-induced dysbiosis-mediated cancer progression (Also refer to Figures S2 and S3) (A–C) Graphical representation of tumor flow-cytometric analysis (left) and bar graphs (right) of control, CSE, antibiotics alone, and CSE + antibiotics group showing percentage infiltration of CD3CD4+ T cells (A), CD3CD8+ T cells (B), and CD11b+Ly6G + MDSC (C) cells of single cells in the tumor microenvironment, respectively, showing CSE-induced immunosuppressive changes reversed with gut microbiome depletion (*n* = 7–10 per group). (D–F) Tumor flow-cytometric analysis showing immunosuppressive changes characterized by decreased CD3CD4+ T cells (D), CD3CD8+ T cells (E), and increased CD11b+Ly6G + MDSC cells (F), respectively, induced in mice that received FMT from NNK-exposed mice vs. controls (*n* = 7–8 per group). (G) Tumor volumes in WT and Rag1-KO mice exposed to CSE with or without gut microbiome depletion with broad-spectrum antibiotics (*n* = 7–10 per group). (H) Tumor volumes in WT and Rag1-KO mice receiving FMT from smoke donors (smoke FMT) vs. control donors (control FMT) (*n* = 9–12 for WT mice, *n* = 8–9 for Rag1-KO mice). (I) Tumor volumes in WT (*n* = 14–15) and CD8-KO mice (*n* = 7–8) exposed to CSE. (J) Tumor kinetics in WT CSE mice with or without Ly6G-depleting monoclonal antibody with appropriate controls (*n* = 10 per group). ∗*p* value <0.05; ∗∗*p* value <0.01; ∗∗∗*p* value <0.001. Unpaired t test or ANOVA used as appropriate for statistical comparison. Data presented as mean ± SEM.

We next characterized the immune tumor microenvironment (TME) of subcutaneous tumors from control and smoke-exposed mice with or without gut microbiome depletion through flow cytometry. Smoke-exposed mice had a globally attenuated anti-tumor immune response with decreased CD3^+^ lymphocytes, CD4^+^ T cells, CD8^+^ T cells, and CD11c+ MHC II + dendritic cells. On the other hand, tumor-promoting moieties, such as myeloid-derived suppressor cells (MDSCs [CD45^+^ CD11b+ Ly6G+]), were upregulated. The M1 (CD45+F4/80+MHCII+CD206-) to M2 (CD45+F4/80+MHCII-CD206+) macrophage ratio was tilted significantly in favor of the M2 phenotype (Figures 2A–2C and S2A–S2C). Gut microbial depletion was able to rescue the anti-tumor immune machinery with downregulation of MDSCs and upregulation of antigen-presenting dendritic cells, tumor-infiltrating CD4^+^ T cells, and CD8^+^ T cells; however, the macrophage polarization could not be reversed (Figure S2C). Intriguingly, this immunomodulation of the TME was also evident in mice receiving FMT from NNK-exposed donors. Immunophenotyping of tumors implanted in NNK FMT recipient mice revealed reduced infiltration of CD3^+^ T cells, CD4^+^ T cells, and CD8^+^ T cells, along with significantly elevated MDSCs (Figures 2D–2F and S2D). Th17 T cells (CD3^+^CD4+IL17+), known to potentiate an immunosuppressive phenotype in the tumor microenvironment, increased with NNK FMT but did not reach statistical significance (Figure S2E). Moreover, tumor growth inhibition seen with therapeutic antibiotic administration to smoke-exposed mice was accompanied by significantly increased CD8^+^ T cell infiltration. MDSCs (CD45^+^CD11b+Ly6G+) infiltration in the TME simultaneously decreased; however, it did not reach statistical significance (Figures S2F and S2G). These findings indicate that gut microbiome depletion rescues the anti-tumor adaptive immune machinery in the TME.

To confirm the importance of the adaptive immune system, we first utilized the Rag1-KO mouse model, which lacks a functional adaptive immune system.^24^ We found that CSE did not promote tumor growth in Rag1-KO mice; additionally, in these mice, gut microbiome depletion failed to decrease the tumor burden (Figure 2G). Administration of NNK instead of CSE to Rag1-KO mice yielded similar results (Figure S2H). This indicates that the adaptive immune system is essential for smoking-induced gut microbial dysbiosis to affect tumor progression.

To eliminate the possibility that potential differences in the baseline microbiome of Rag1-KO mice, when compared to the WT mice, could be confounding our results, we reconstituted the gut-microbiome-depleted Rag1-KO mice with FMT from either smoke-exposed or smoke-free WT mice. The tumor-promoting effect of the smoke-exposed dysbiotic gut microbiome was abrogated in Rag1-KO mice (Figure 2H). To specifically delineate the role of the CD8 adaptive immune arm, we evaluated the effect of smoking in CD8-KO mice and found CSE did not promote tumor growth, signifying that the effector immune cells are essential links for the dysbiotic microbiome to mediate its effects (Figure 2I). To test whether antibiotics treatment led to increased cytotoxicity in CD8^+^ T cells, we performed an *ex vivo* cytotoxicity study by incubating splenic CD8^+^ T cells, isolated from tumor-bearing mice, with calcein-AM-labeled KPC cells (see STAR Methods). CD8^+^ T cells isolated from CSE mice treated with antibiotics had significantly greater cytotoxicity against KPC cells *ex vivo*, as compared to CSE mice without antibiotics (Figure S3A). Additionally, Ki67 staining of tumors obtained from CSE + antibiotics mice demonstrated less cancer cell proliferation as compared to CSE mice (Figure S3B), indicating that increased CD8^+^ T cell infiltration and cytotoxicity may be leading to decreased cancer cell proliferation. As smoke-exposed tumors showed increased accumulation of CD11b+Ly6G + cells, which have been previously shown to decrease cytotoxic CD8^+^ T cell infiltration in the TME,^25^ we hypothesized that these cells were responsible for decreased effector CD8 T cell tumor infiltration in smoke-exposed mice. Specifically targeting these cells using anti-Ly6G monoclonal antibody (Invivomab Catalog# BE0075-1, Clone 1A8), with cell depletion confirmed on flow cytometry of splenocytes (Figure S3C), prevented the tumor-promoting effects of smoking (Figure 2J).

### Smoke-induced dysbiosis is characterized by unique microbial and metabolomic signatures amenable to selective targeting

Gut microbial profiling by 16S rRNA sequencing revealed that the CSE group clustered separately compared to the control group upon principal coordinate analysis (PCoA) (Figure S4A). Linear regression analysis showed significant enrichment of gram-negative genera (*Bacteroides* and *Akkermansia)* and certain gram-positive genera (*Turicibacter, Acetatifactor,* and *Blautia)* in the CSE group (Figure S4B). Similarly, NNK exposure induced a significant difference in beta diversity (Figure S4C), and interestingly, gram-negative genera like *Bacteroides and Akkermansia* were enriched upon NNK exposure as well (Figure S4D).

**Figure 3:**
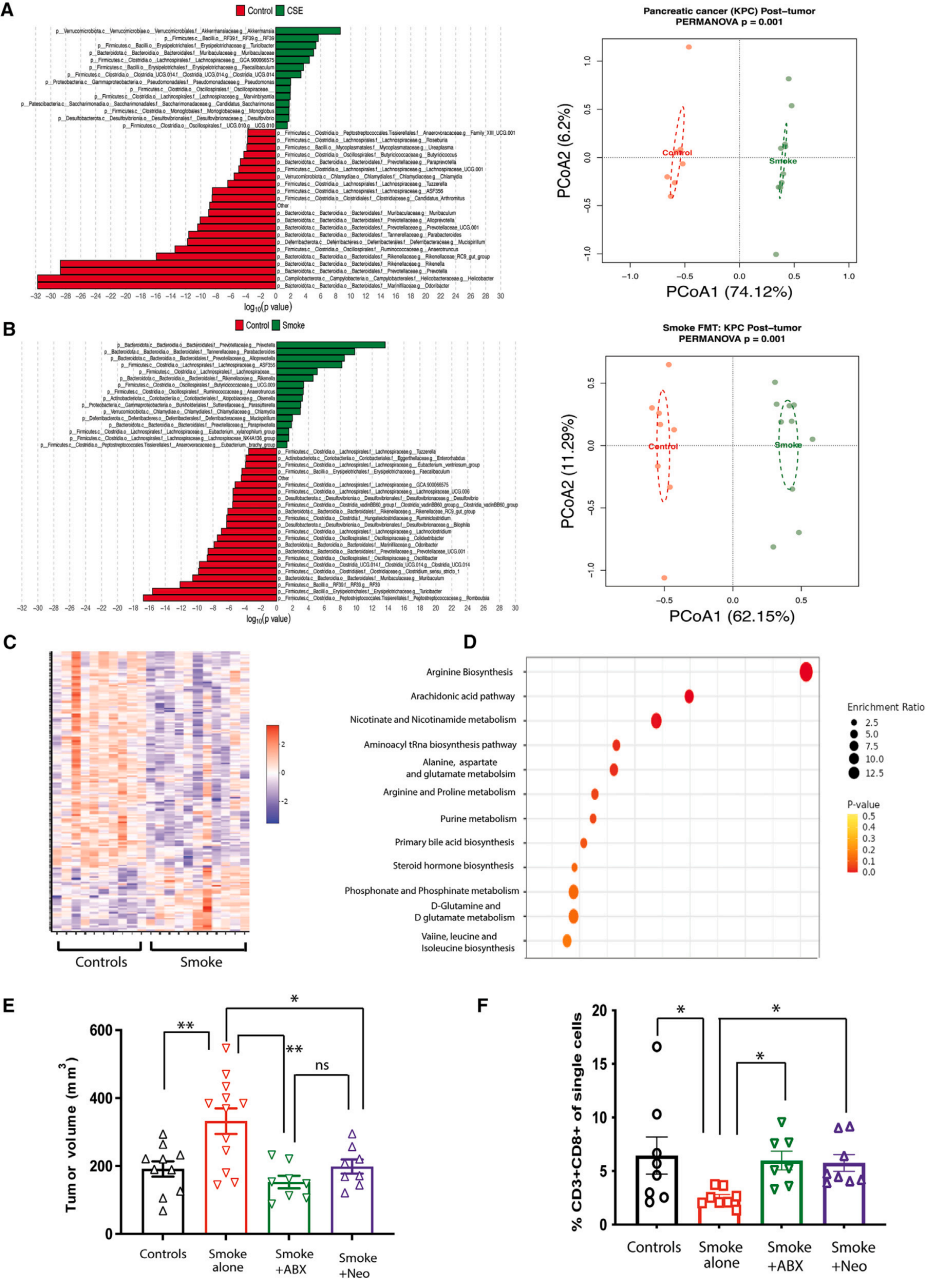
Smoke-induced dysbiosis is characterized by unique microbial and metabolomic signatures amenable to selective targeting (Also refer to Figures S4 and S5) (A) Fecal samples were obtained from subcutaneous KPC tumor-bearing control and CSE mice and analyzed using 16s rRNA amplicon sequencing for metagenomic characterization. (Left panel) The significantly enriched microbes based on linear model analysis in the CSE (green bars) and control (red bars) groups are shown. (Right panel) PCoA plot comparing beta diversity between control (red) and CSE (green) groups. Distance was calculated using Bray-Curtis analysis, and PERMANOVA was used for statistical significance. (B) Fecal samples obtained from C57BL/6J mice receiving FMT from control or CSE donors and implanted with KPC pancreatic tumors were similarly interrogated through 16 s rRNA sequencing. Linear model (left panel) and Bray-Curtis analysis (right panel) are shown. (C) Heatmap representation of all differential fecal metabolites (BH FDR <0.25) between tumor-bearing control (*n* = 10) and CSE (*n* = 11) mice. A total of 194 differential metabolites were obtained upon untargeted LC-MS analysis. (D) Significantly altered metabolic pathways were obtained using pathway enrichment analysis of significantly altered fecal metabolites upon CSE using MetaboAnalyst 5.0. Only the metabolites annotated in HMDB were used for the analysis (88/194 differential metabolites). (E) Subcutaneous tumor volumes at endpoint after CSE with selective targeting of gut microbiome using oral neomycin gavage (200 mg/kg) or complete gut microbiome depletion using broad-spectrum antibiotics cocktail (*n* = 8–12 per group). (F) Flow cytometric analysis of tumors showing % of CD3^+^CD8^+^ of single cells after selective targeting of gut microbiome using oral neomycin gavage (200 mg/kg) or broad-spectrum antibiotic cocktail (*n* = 7–8 per group). ∗*p* value <0.05, ∗∗*p* value <0.01. CSE, cigarette smoke exposure; PCoA, principle coordinate analysis; PERMANOVA, permutational analysis of variance; FMT, fecal microbiome transplant; LC-MS, liquid chromatography-mass spectrometry; BH, Benjamini-Hochberg; FDR, false discovery rate; HMDB, human metabolome database. Unpaired t test or ANOVA used, as appropriate, for statistical comparison. Data presented as mean ± SEM.

When the gut microbiome composition of mice bearing subcutaneous tumors was evaluated, the CSE group again demonstrated a distinct gut microbial signature. In KPC subcutaneous tumor-bearing mice, bacteria belonging to *Akkermansia* spp., *Turicibacter* spp., *Faecalibaculum* spp., *and Pseudomonas* spp. were the most enriched in the CSE group (Figure 3A). Furthermore, CSE led to a significantly different gut microbial composition in mice bearing subcutaneous MC-38 colon cancer (Figure S4E) and MB-49 bladder tumors (Figure S4G). Interestingly, despite a different tumor microenvironment, bacteria from genus *Akkermansia* were the enriched bacteria in the gut in the CSE group in both tumor types, similar to KPC pancreatic tumors (Figures S3F and S4H). Additionally, tumor-bearing mice that were receiving smoke FMT had significant enrichments of bacteria from the gram-negative genera *Prevotella*, *Parabacteroides*, and *Alloprevotella* compared to mice that received control FMT (Figure 3B).

To evaluate the gut microbial metabolomic signature induced by CSE, we performed untargeted liquid chromatography-mass spectrometry (LC-MS) metabolomics on fecal samples from tumor-bearing control and CSE mice. A total of 781 unique metabolites were identified. Unbiased hierarchical clustering using a scale-normalized concentration of discriminatory metabolites with a false discovery rate <0.25 in control and smoke-exposed groups showed that the two groups had distinct signatures, with 194 metabolites having a significantly differential abundance (Table S1; Figure 3C). Qualitative metabolic set enrichment analysis of metabolites that were significantly altered in the CSE group compared to the controls revealed that arginine biosynthesis and arachidonic acid metabolism were the top metabolic pathways that were altered due to CSE (Figure 3D).

Since CSE led to the enrichment of both gram-positive and gram-negative bacterial species, we utilized selective targeting of these microbes using narrow-spectrum antibiotics. Neomycin, which has a predominantly gram-negative spectrum, significantly reduced tumor burden in CSE mice. In contrast, vancomycin (exclusively gram-positive spectrum) showed only partial tumor reduction (Figure S5A). Additionally, neomycin demonstrated an anti-tumor effect equivalent to the broad-spectrum antibiotic cocktail (Figure 3E). Neomycin treatment partially ameliorated the metabolomic changes induced due to CSE (Figures S5B and S5C). Finally, selective targeting with neomycin also reverses the immunosuppressive changes in the TME, as evidenced by increased CD8^+^ T cell tumor infiltration (Figure 3F).

## Discussion

We report one of the first studies demonstrating the role of gut microbiome in propagating pro-tumorigenic effects of CSE across multiple smoking-dependent cancers. CSE was associated with an immunosuppressive TME, and its effect was neutralized when the gut microbiome was depleted. FMT experiments confirmed the tumor-promoting and immunosuppressive effects of this CSE-modulated gut microbiome. Our results are in line with a recent study by Bai et al., where cigarette smoke expedited colon cancer growth in an azoxymethane-induced colitis model through enrichment of *Eggerthella lenta.*^26^ Prior pre-clinical investigations have focused on alterations in cell signaling pathways, such as nicotinic receptor stimulation or genetic/epigenetic changes in proto-oncogenes and tumor suppressor genes, as mechanistic explanations for smoking-induced carcinogenesis. However, in our study, FMT experiments suggest that CSE-induced gut microbial dysbiosis can independently exert tumor-promoting effects. Moreover, ablation of the gut microbiome in mice receiving CSE led to abrogation of tumor growth, whereas the levels of smoke metabolites in serum (cotinine) or stool (polyaromatic hydrocarbons) were unaffected. This suggests that even in the presence of circulating CSE components, the gut microbiome plays an essential role in promoting tumor growth.

Exposure to smoke, or FMT from CSE mice, led to significant upregulation of granulocytic MDSCs in the TME, and this immunosuppressive phenotype was reversed with gut microbiome depletion. In addition, MDSC-specific marker antibody depletion was able to reverse the tumor-promoting effects of smoking, implicating a possible mechanistic link between the dysbiotic gut microbiome induced by CSE and myeloid cell infiltration. Interestingly, Pushalkar et al. similarly demonstrated that the gut microbiome can modulate MDSC infiltration in the TME in KC genetic model of pancreatic cancer.^19^ Another dominant immunosuppressive population in pancreatic cancer TMEs is tumor-associated macrophages.^27^ Prior work from Kumar et al. indicates that smoke exposure can induce differentiation of Ly6G + MDSCs into tumor-associated macrophages (TAMs), which secrete epidermal growth factor receptor (EGFR) ligands to accelerate PanIN development in the KC mouse model.^28^ Similarly, we found increased M2-polarized macrophages (F4/80+, MHC II−, CD206 +) in the TME upon CSE; however, this could not be reversed upon gut microbial ablation, indicating that macrophage polarization likely does not explain the effects of gut microbial targeting on tumor growth. Additionally, CSE significantly impacted adaptive immune response in the TME and decreased CD8 T cell infiltration. These findings are concordant with recent human studies in esophageal, head, and neck squamous cell cancer, where smokers had decreased CD8 T cell infiltration in the TME.^29^^,^^30^ Treatment with antibiotics was able to increase CD8^+^ T cell infiltration in the TME as well as CD8^+^ T cell cytotoxicity on *ex vivo* analysis. The loss of pro-tumorigenic effects of CSE in Rag1-KO and CD8-KO mice, along with the failure of smoke-FMT to increase tumor growth in Rag1-KO mice, suggests the need for functional adaptive immunity for CSE dysbiosis to promote tumor growth.

Characterization of the CSE dysbiotic gut microbiome through 16s rRNA sequencing revealed tumor-specific enrichment patterns. However, certain microbes, such as *Akkermansia* spp. and the *Clostridiales vadin* BB60 group, were consistently elevated across tumor types. *Akkermansia* has been favorably associated with immunomodulatory responses in obesity and homeostatic immunity but also has been pathologically indicated in disease states like multiple sclerosis.^31^^,^^32^^,^^33^^,^^34^ The gut microbiome is known to exert its effects in a context-specific manner.^10^ It is possible that under the influence of CSE, *Akkermansia* assumes a pro-tumorigenic phenotype. However, further experiments are needed to substantiate such conclusions. We did observe significant anti-tumor activity with neomycin treatment that might indicate a specific role for gram-negative dysbiosis in smoke-mediated tumor progression. There have been recent reports regarding accumulation of nicotine in the intestinal lumen and its degradation by gut bacteria in the context of NASH cirrhosis.^23^ Interestingly, in our study, although nicotine levels in the luminal contents of ileum increased with smoke exposure, we did not observe any differences in nicotine levels upon antibiotics treatment. This combined with unchanged serum cotinine levels in smoke-exposed mice upon antibiotics treatment may indicate that in our model, nicotine metabolism by gut bacterium did not play a significant role. This discrepancy may reflect the different biological context, when comparing NASH cirrhosis to implanted cancer models. However, further studies are needed to establish whether nicotine metabolism by gut microbiome is essential for tumor-promoting effects of cigarette smoke.

CSE also led to alterations in composition of stool metabolites that are known to exert immunomodulatory effects. CSE was associated with significantly altered arachidonic acid metabolism, which was partially reversed through neomycin treatment. The arachidonic acid pathway can significantly affect the induction of inflammation in both benign and malignant disease states. Prostaglandin E2 can induce profound immunosuppression via various mechanisms, including infiltration of MDSCs in the TME,^35^ whereas leukotriene B4 is a potent myeloid chemoattractant.^36^^,^^37^ We also observed significant alterations in the arginine biosynthesis pathway on CSE. L-arginine is essential for T lymphocytes to mount an anti-tumor immune response,^38^ and depletion of extracellular arginine by MDSC-expressed arginase I can lead to immunosuppression in the tumor microenvironment.^39^ Interestingly, our data indicate downregulation of L-arginine upon CSE and subsequent increase upon neomycin treatment. Bai et al.^26^ identified alterations in bile-acid metabolism that could explain the effects of smoking-induced dysbiosis on colon cancer progression. In their experiments, taurodeoxycholic acid (TCDA), a secondary bile acid, was upregulated upon smoke exposure, and the authors posited that this could promote cancer growth through the MAP/ERK signaling pathway. In our stool metabolomic profiling, we did not observe an enrichment of bile acid metabolism in CSE mice. The highly context-specific composition and phenotypic effects might explain the differences in the microbes and their metabolites identified in our studies. For example, in our study, antibiotics treatment alone decreased the tumor burden in the KPC model but did not affect the tumor burden in MC-38 or MB-49 models. Regardless, antibiotics treatment consistently decreased the tumor-promoting effects of CSE in all these models.

In summary, we demonstrate the induction of a unique metabolic and microbial signature upon smoke exposure that creates an immunosuppressive tumor microenvironment and favors cancer progression.

### Limitations of the study

Our study does have certain limitations. It is unclear how smoking-induced dysbiosis is established and what mechanisms underlie the interaction between the dysbiotic gut microbiome and anti-cancer immune response. Whether these effects are mediated by specific microbes or a group of microbes sharing a specific functional phenotype needs to be clarified. For example, we did notice a preponderance of gram-negative bacteria upon CSE; however, the mechanisms by which these bacteria interact with and modulate the innate and/or adaptive immune system has not been explored in our study. Additionally, the role of immunomodulatory metabolic pathways altered upon CSE needs to be investigated.

## Resource availability

### Lead contact

Further information and requests for resources should be directed to and will be fulfilled by the lead contact, Vikas Dudeja (vdudeja@uabmc.edu).

### Materials availability

No new unique reagents were generated in this study.

### Data and code availability

• Data: the metabolomics data raw files can be accessed through NIH metabolomics workbench using Data track ID 4229 and Study ID is ST002823. The 16 S rRNA raw sequencing files can be accessed using the NCBI Bio project accession number PRJNA1007862. The following link can be used to access the data after the release date. https://www.ncbi.nlm.nih.gov/sra/PRJNA1007862.
• Code: the code used for processing and analyzing the 16s rRNA data can be accessed at https://github.com/asorgen/Smoking_Cancer_Gut_Dysbiosis_Analysis_2023.git. All analysis was done using R (https://www.r-project.org/).
• All other data are available upon request from the lead contact.

## Acknowledgments

The authors would like to thank the Department of Veterinary Resources for help with the animal experiments at the University of Alabama and University of Miami. This work was supported by the following grants to V.D.: 10.13039/100000005DOD grants W81XWH-20-1-0738 and W81XWH-21-1-0649, 10.13039/100000002NIH R01 DK 111834, I01CX002478-01A1 and I01BX004944 from 10.13039/100019592Veterans Administration and FL James and Esther King Biomedical Research Award 9JK07 from Florida Department of Health. The metabolomics core was supported by the Cancer Prevention and Research Institute of Texas (CPRIT) Core Facility Support Award RP210227 "Proteomic and Metabolomic Core Facility," NCI Cancer Center Support Grant P30CA125123, 10.13039/100000002NIH/NCI R01CA220297, and 10.13039/100000002NIH/NCI R01CA216426 intramural funds from the 10.13039/100008527Dan L. Duncan Cancer Center (DLDCC). The graphical abstract was created with Biorender.com.

## Author contributions

Conception, P. Sharma, T.J., S.K., and V.D.; experiments, P. Sharma, T.J., A.S., S.I., M.T., P.R., S.K., V.S., E.P.B., A.K.G.-G., U.V., D.S.S., D.E., J.A., K.R.K.R., C.S.A., and A.H.M.K.; review of literature, P. Sharma, T.J., A.S., S.K., V.S., C.S.A., and A.H.M.K.; acquisition and validation, P. Sharma, T.J., A.S., A.K.G.-G., D.S.S., P. Sahay, D.E., J.A., K.R.K.R., C.S.A., A.H.M.K., A.F., and V.D.; writing and editing, P. Sharma, T.J., A.S., S.P., K.R.K.R., A.H.M.K., A.F., and V.D.; agreement and accountability for integrity of work, all authors.

## Declaration of interests

The authors report there are no competing interests to declare.

## STAR★Methods

### Key resources table

**Table:** 

REAGENT or RESOURCE	SOURCE	IDENTIFIER
**Antibodies**		

FC: anti-mouse CD45	Biolegend	Clone:30-F11, Cat 103106; RRID:AB_312971
FC: anti-mouse CD3e	Biolegend	Clone:145-2C11 Cat 100348; RRID: AB_2564028
FC: anti-mouse CD4	Biolegend	Clone: RM4-5 Cat 100528; RRID: AB_312729
FC: anti-mouse CD8	Biolegend	Clone:53–6.7 Cat 100734; RRID: AB_2075239
FC: anti-mouse Ly6G	Biolegend	Clone: 1A8 Cat 127624; RRID: AB_10645331
FC: anti-mouse CD11b	Biolegend	Clone: M1/70 Cat 101228; RRID: AB_893232
FC: anti-mouse MHC II	Biolegend	Clone: M5/114.15.2 Cat 107622; RRID: AB_493727
FC: anti-mouse F4/80	Biolegend	Clone: BM8 Cat 123110; RRID: AB_893486
FC: anti-mouse CD25	Biolegend	Clone: PC61 Cat 102024; RRID: AB_493709
FC: anti-mouse CD44	Biolegend	Clone: 1M7 Cat 103040; RRID: AB_10895752
FC: anti-mouse CD11c	Biolegend	Clone: N418 Cat 117318; RRID: AB_493568
FC: anti-mouse TNF-a	Biolegend	Clone: MP6-XT22 Cat 506333; RRID: AB_2562450
FC: anti-mouse CD206	Biolegend	Clone: C068C2 Cat 141710; RRID: AB_10900445
FC: anti-mouse CD62L	Biolegend	Clone: MEL-14, cat 104445; RRID: AB_2564215
FC: anti-mouse IL-17 A	Biolegend	Clone: TC11-18H10.1 Cat 506926; RRID: AB_10900442
IF: Anti-mouse-Krt19	Abcam	Clone: EP1580Y Catalog Ab52625; RRID: AB_2281020
IF: Anti-Mouse-Ki67	Invitrogen	Clone: SP6 Catalog MA5-14520; RRID: AB_10979488

**Chemicals, peptides, and recombinant proteins**		

NNK	Sigma	*N*-076-1ML

**Critical commercial assays**		

Serum cotinine assay	Calbiotech	Cat#CO096D
Calcein AM	Invitrogen	C3100MP

**Deposited data**		

16s rRNA sequencing data	Mouse stool	NCBI Bio project accession number PRJNA1007862https://www.ncbi.nlm.nih.gov/sra/PRJNA1007862
untargeted LC-MS metabolomics	Mouse stool	NIH metabolomics workbench Data track ID 4229 and Study ID is ST002823

**Experimental models: Cell lines**		

KPC pancreatic cancer cell line	In-house breeding	N/A
MC-38	ATCC	Cat# CRL-2639
MB-49	Sigma	Cat# SCC148

**Experimental models: Organisms/strains**		

C57BL/6J mouse	Jackson Laboratory	000664
Rag1 KO mouse	Jackson Laboratory	002096
CD8 KO mouse	Jackson Laboratory	002665

**Software and algorithms**		

Flow Jo	Tree Star	V10.0
R	The R project	https://www.r-project.org/
Metaboanalyst	https://genap.metaboanalyst.ca/	v5.0
Code for 16s rRNA analysis	This manuscript	https://github.com/asorgen/Smoking_Cancer_Gut_Dysbiosis_Analysis_2023.git

### Experimental models and subject details

#### Animal models

Animal experiments were authorized and overseen by the Institutional Animal Care and Use Committee (IACUC) and performed in accordance with approved protocols (UAB APN 22162 and University of Miami APN 18–142). C57BL/6J WT mice, A/J mice (stock no. 000646), Rag1-KO mice (B6.129S7-*Rag1*^*tm1Mom*^/J), as well as CD8-KO mice (B6.129S2-*Cd8a*^*tm1Mak*^/J), were purchased from the Jackson Laboratory (Bar Harbor, ME). *LSL-Kras*^*G12D/+*^*;Pdx-1-Cre* (KC) mice were generated in our breeding facility by crossing *LSL-Kras*^*G12D*^ mice with *Pdx-1cre* mice. Mice were backcrossed to the C57BL/6J genetic background for at least ten generations. Experiments with WT, Rag1-KO, and CD8-KO were performed with 6–8 weeks old female mice. Mice of both sexes were recruited for KC mice experiments and A/J mice. Littermates of genetically modified mice were used as controls.

#### Smoke chamber

The TE-10 smoking machine (Teague Enterprises) was used to provide smoke exposure to mice. Humidified 3R4F cigarettes (Tobacco Health Research Institute, Lexington, KY) were used to provide cigarette smoke. A smoke concentration of 150–200 mg/mm^3^ was maintained inside the chamber, and carbon monoxide levels were monitored. Mice were acclimated to cigarette smoke during the first week through incremental increases in smoke exposure from 1 h/day to 4 h/day. This level of exposure was continued for the duration of the experiment. Mice kept in a compartment with room air flow instead of cigarette smoke were used as controls.

#### *In vivo* experiments

For smoke chamber experiments with WT mice, CD8-KO mice, or Rag1-KO mice, 6–8-week-old female mice were utilized. For antibiotic depletion experiments (using WT mice or Rag1-KO mice), mice were divided into four conditions - control mice, mice with smoke exposure alone, mice receiving antibiotics alone, and mice receiving smoke exposure along with antibiotics. The pre-exposure phase was four weeks, during which mice were given cigarette smoke and/or antibiotics. Subsequently, the mice were challenged with subcutaneous tumors. We first standardized the pre-exposure and found that four weeks was sufficient time to develop the tumor-promoting effects of smoke. The broad-spectrum antibiotics included - vancomycin (100 mg/kg), ampicillin (200 mg/kg), metronidazole (200 mg/kg), neomycin (200 mg/kg), and amphotericin (1 mg/kg). Ampicillin was dissolved in drinking water, while the rest were dissolved in phosphate buffered saline (PBS) and administered as a daily gavage of 0.5 mL solution. Smoke and antibiotics were continued after the tumor challenge until the experimental endpoint. For the CD8-KO mice experiment, WT and CD8-KO mice were divided into two groups each - control mice and smoke-exposed mice. The smoke pre-exposure period lasted four weeks, after which mice were implanted with subcutaneous tumors. Exposure was continued until the endpoint. To test the effects of antibiotics in a therapeutic setting, WT mice were initially divided into control mice and smoke-exposed mice. After the pre-exposure phase, mice were challenged with subcutaneous tumors, which were allowed to grow for two weeks. Both groups were further randomized into two groups - one without an antibiotic cocktail and one with an antibiotic cocktail. For anti-Ly6G experiment, control and CSE mice were pre-exposed for 4 weeks and subsequently randomized to treatment with anti-Ly6G mAb (Invivomab Catalog# BE0075-1, Clone 1A8) or isotype control. Mice were treated with 250μg/dose two times a week starting day 1 post tumor implantation.

For NNK experiments, a setup similar to the smoke chamber was used. Four groups were as follows - control mice, NNK alone, antibiotics alone, and NNK and antibiotics. NNK pre-exposure was given for six weeks through weekly injections, i.p 100 mg/kg. This was followed by a subcutaneous tumor challenge, and injections of NNK were continued until the endpoint. For the KC mice experiment, 8-week-old KC mice were randomly recruited to each of the four groups mentioned above. Weekly NNK injections and/or antibiotic cocktails were given for eight weeks, after which mice were euthanized at four months of age. A/J mice were given weekly NNK injections from 6 to 10 weeks of age, euthanized at 28 weeks, and individual tumor nodules in the lungs were recorded.

For FMT experiments, stool was collected from cancer-naive control or smoke-exposed mice (4 weeks). The pellets were homogenized and dissolved in PBS. Eight to ten pellets were dissolved in 10 mL PBS. The mixture was then centrifuged at 500g for 5 min to allow fecal matter to settle down. The supernatant was collected and administered to recipient mice through oral gavage (500μL). The recipient mice were prepared for FMT by ablating their gut microbiome using a broad-spectrum antibiotic cocktail, as already described, for two weeks. A washout period of one week was allowed for the antibiotics to flush out of the system. This was followed by biweekly oral gavage of fecal microbiome from control or smoke-exposed mice for four weeks to reconstitute the microbiome. Subcutaneous tumors were then implanted, and biweekly FMT continued until the endpoint.

#### Cell lines

The KPC cell line was derived from *LSL-Kras*^*G12D/+*^*;LSL-Trp53*^*R172H/+*^*;Pdx-1-Cre* (KPC) mice being maintained in our breeding colony. Briefly, tumor fragments obtained from resected pancreatic specimens of 6 months old female KPC mouse were plated in 10% DMEM. Magnetic selection and differential trypsinization were used to separate outgrowing cancer cells from fibroblasts. Mycoplasma testing was done using qPCR based assay at Charles River Laboratories (Mouse Comprehensive Clear Panel). MC-38 colon cancer cell line (ATCC Number: CRL-2639) were obtained from ATCC while MB-49 bladder cancer cell line was obtained from Sigma (Cat no. SCC148). All cell lines were passed in 10% DMEM with 1% Penicillin-Streptomycin. Cell lines from passages 5–15 were used for *in vivo* experiments.

### Method details

#### Flow cytometry

Single cell suspensions of subcutaneous tumors were made after digestion using collagenase type IV (Worthington Biochemical Corp., Catalog # LS004188). Cells were washed with a solution containing phosphate buffered saline (PBS) with 1% Bovine Serum Albumin (Catalog Number Catalog #: A9418-100G, Sigma Aldrich) after which staining was done for surface markers. This was followed by cell fixation using a fixation buffer (BD Pharmingen, Catalog # 505034). Subsequently, cells were permeabilized using eBioscience Permeabilization Buffer (10X) (Thermo Fisher, catalog 00-8333-56) and were stained for intracellular markers. Acquisition was done using the LSR Fortessa II (BD Biosciences, NJ) and data were analyzed with the help of FlowJo software (Tree Star, Ashland, OR). A list of antibodies is provided in Table S2.

#### Serum cotinine assay

Serum samples obtained were analyzed using a serum cotinine elisa kit (Calbiotech, Cat#CO096D). The samples were then processed following the manufacturer’s protocols. All samples were plated in duplicates. Absorbance was measured at 450nm.

#### Histology and immunofluorescence

For histological analysis, pancreatic specimens were fixed in formalin, dehydrated in ethanol, embedded with paraffin and stained with H&E. For Immunofluorescence analysis, slides containing tissue sections were baked for 2 h at 60°C. Then tissue sections were deparaffinized using xylene, rehydrated with decreasing concentrations of ethanol and permeabilized by incubating in 0.1% Triton X- for 10 min. After antigen retrieval and blocking in 5% normal goat serum, incubated with primary antibody (Abcam Rb Antimouse/human Krt19, Catalog # Ab52625; Invitrogen Rb Antimouse Ki67, Catalog# MA5-14520) overnight at 4^0^C. After washing in PBS, tissue sections were incubated with fluorophore conjugated secondary antibody (Life Technologies, AF 594 donkey anti-Rb IgG, Catalog #R37119) for 1 h at room temperature. Slides were washed in PBS and mounted with Vectashield containing 4′ 6′-diamidino2-phenylindole (DAPI). Fluorescent images were captured using a confocal microscope (Nikon) with standardized settings. The images were quantified using ImageJ software using a blind morphologist (NIH). For quantification of each mouse pancreas % area stained/Low power field was calculated using 5 Low power fields that covered the entire slide.

#### Ex-vivo cytotoxicity assessment with CalceinAM assay

CD8^+^ T-cells were extracted using MACS sorting (130–104-075; Miltenyi Biotec) from spleens of tumor bearing mice exposed to room air or CSE +/− antibiotics cocktail and subjected to Calcein AM (C3100MP; Invitrogen) based cytotoxicity assay. The target tumor cells, KPCs were stained with Calcein, and co-cultured with CD8^+^ T-cells (effectors) in Effector: Target ratio of 20:1. 10^6^ KPCs/mL were labeled by preparing a 2 μg/mL Calcein AM staining media in complete DMEM F-12 medium and incubated for 30 min at 37°C with intermittent mixing. 5000 labeled KPCs and 1x10^5^ CD8^+^ T-cells were aliquoted in a single well of a 96 well plate in triplicates. Maximum release and spontaneous release controls were set up by adding 1% Triton X-100 and plain media, respectively, to wells containing labeled 5000 kPCs. The plate was incubated for 4 h at 37°C in 5% CO2, centrifuged and 150 μL of the supernatant was transferred to a flat bottom black plate. The fluorescence was read at Ex: 485 nm/Em: 530 nm employing Infinite M200 plate reader (Tecan). The percent cell lysis was calculated using the following formula and plotted.

[(Test Release−Spontaneous Release)/(Maximum Release−Spontaneous Release)] x 100.

#### Microbial 16S rRNA sequencing

Stool samples from the distal rectum were collected. All samples were snap frozen in liquid nitrogen vapor, and then equal amounts were used for further DNA collection using the PowerSoil DNA Isolation Kit (Mo Bio Labs, Carlsbad, CA) according to the manufacturer’s instructions. 16S ribosomal RNA gene sequencing from stool samples was done at the University of Minnesota Genomic Core. Template DNA quantity in samples was determined by creating 1:8 and 1:64 dilutions of each sample and subjecting them all to qPCR using primers V4_515F_Nextera and V4_806R_Nextera targeting the V4 locus in the rRNA gene. Samples were diluted with water to normalize starting template abundances, and then amplicons were generated by subjecting sample DNA to 25-cycle PCRs using the same primers. PCR products were diluted 1:100 in water and subjected to a second 10-cycle PCR to attach Illumina sequencing primer-compatible DNA regions as well as individual barcodes for each sample. Samples were all uniquely dual-indexed, as detailed in Gohl et al.^40^ Final PCR products were normalized using SequalPrep kits (Invitrogen), pooled into sequencing libraries, and cleaned with AMPure XP mag beads (Beckman Coulter). Sequencing libraries were loaded onto an Illumina MiSeq using a 2x300 v3 flow cell (Illumina, San Diego, CA).

#### Metabolomics

Total metabolites were extracted from fecal samples using the liquid-liquid extraction method and analyzed through high-throughput liquid chromatography-mass spectrometry (LC-MS/MS) as described previously.^41^^,^^42^ For untargeted metabolomics, mass spectrometry data were acquired in both positive and negative ionization modes via the data-dependent acquisition (DDA) method using a TripleTOF 5600 mass spectrometer equipped with a Turbo VTM ion source (AB Sciex, Concord, Canada) coupled to Shimadzu CTO-20A Nexera X2 UHPLC system (Shimadzu, Maryland, USA). The details of the LC-MS/MS methods are described earlier.^41^^,^^42^ The raw data were converted to mgf as described previously.^43^ Identified peaks were carefully reviewed using MultiQuant software (ver. 1.1.0.26, AB Sciex, Concord, Canada). The relative peak area was log2 transformed, followed by internal standard normalization for each method. The differentially expressed analysis was applied with the Benjamini-Hochberg method^44^ for false discovery rate (FDR <0.25) correction accounting for multiple comparisons.

#### Ileal nicotine estimation

For measurement of nicotine, ileal luminal contents were collected and snap frozen in liquid nitrogen at experimental endpoint. The analysis was conducted using high-throughput liquid chromatography paired with tandem mass spectrometry (LC-MS).^41^^,^^42^^,^^43^ For chromatographic separation, Hydrophilic Interaction Chromatography (HILIC) was applied with an XBridge Amide column (3.5 μm, 4.6 × 100 mm, Waters, Milford, MA). Mobile phases A and B consisted of 0.1% formic acid in water and acetonitrile, respectively, following a gradient: 0–2 min at 85% B; 2–5 min at 30% B; 5–7 min at 2% B; 1 min at 95% B, before re-equilibrating to the initial 85% B condition over 10 min. The flow rate was set to 0.3 mL/min, with a column temperature of 50°C and an injection volume of 10 μL.

Data acquisition used multiple reaction monitoring (MRM) with a 6495B Triple Quadrupole mass spectrometer connected to a 1260 Infinity II HPLC system (Agilent Technologies, Santa Clara, CA) and managed through Agilent Mass Hunter Software. The mass spectrometer operated in electrospray positive mode, with source parameters set to a Gas Temperature of 250°C, Gas Flow of 14 L/min, Nebulizer pressure of 20 psi, Sheath Gas Temperature of 350°C, Sheath Gas Flow of 12 L/min, Capillary voltage of 3000 V, and Nozzle Voltage of 1000 V. Mass spectra were processed using Agilent Mass Hunter Quantitative Analysis Software, where each compound peak was integrated and normalized against a spiked internal standard. The data were then log2-transformed for further analysis.

### Quantification and statistical analysis

For each animal experiment, n represents number of animals used in the experiment. For each experiment analysing tissue obtained from animals (e.g., flow cytometry, metabolomics, IHC), each data point reflects tissue obtained from one animal. If multiple experiments were performed, data from a representative experiment is presented in the manuscript, with figure legend indicating how many independent experiments were performed. Two groups comparisons and multigroup comparisons were done using unpaired t test and Analysis of variance (ANOVA) respectively using Graph pad prism 9. Data is presented as mean ± SEM. Further details for each specific experiment can be found in figure legends and results section.

#### Microbial 16S rRNA analysis

The Quantitative Insights Into Microbial Ecology (QIIME2; v. 2019.1) pipeline was used to process all 16S rRNA microbial DNA sequences.^45^ Sequences were paired, trimmed, and denoised using the DADA2 plug-in to generate amplicon sequence variants.^46^ Any amplicon sequence variants with a frequency less than ten were filtered out, and open-reference operational taxonomic unit (OTU) clustering was conducted using a 99% sequence identity threshold to the SILVA 138 database.^47^ *De novo* chimeric sequences are identified and removed using the UCHIME algorithm. The resulting OTU table was further filtered to remove rare OTUs (with a relative abundance of <0.01%) and samples with low read counts (<10,000 reads). Consensus sequence taxonomic classification was performed with VSearch and the SILVA 138 taxonomy reference base. Per-sample read count normalization was performed according to the following formula to account for differences in sequencing depth among all samples:

Taxa present in less than 10% of samples were removed from linear regression analyses to avoid the detection of stochastic differences in rare taxa and to maintain power during multiple hypothesis testing using the Benjamini-Hochberg False Discovery Rate procedure.^44^ Beta diversity was determined using Bray-Curtis dissimilarity distances, and ordinations were plotted using the Principal Coordinate Analysis "capscale" function in the Vegan package in R.^48^ The statistical significance of sample groupings based on Bray-Curtis dissimilarity was calculated using Permutational ANOVA testing.

#### Metabolomics analysis

Metabolomics data was analyzed using Enrichment Analysis in MetaboAnalyst 5.0.^49^ Differential metabolites with Benjamini-Hochberg FDR <0.25, which were well-annotated in the Human Metabolome Database,^50^ were used in the analysis. Therefore, a total of 88 metabolites were used in the final analysis (Table S1). The resulting enriched Kyoto Encyclopedia of Genes and Genomes (KEGG)^51^ human metabolic pathways containing at least two entries were generated. Unbiased hierarchical clustering of control and smoke-exposed fecal samples based on differential metabolites and its graphical representation through heatmap was performed using the R package "heatmaply."

## Supplemental information

Document S1. Figures S1–S5 and Table S2

Table S1. Differential metabolites between control and smoke-exposed mice, also refer to Figure 3C **ST1A –** List of differential metabolites between control and smoke-exposed mice with FDR <0.25. **ST1B –** Annotation of metabolites for qualitative metabolic set enrichment analysis in MetaboAnalyst. Metabolites that were not annotated were not used for analysis.
