# Supplementary material for: Smoking-induced gut microbial dysbiosis mediates cancer progression through modulation of anti-tumor immune response
Source: iScience. 2025 Feb 11;28(3):112002. doi: 10.1016/j.isci.2025.112002 (PMC11914281; doi:10.1016/j.isci.2025.112002)
Supplement: Document S1. Figures S1–S5 and Table S2 [file mmc1.pdf]

iScience, Volume *28*

Supplemental information

Smoking-induced gut microbial dysbiosis mediates cancer progression through modulation of anti-tumor immune response

Prateek Sharma, Tejeshwar Jain, Ali Sorgen, Srikanth Iyer, Mohammad Tarique, Pooja Roy, Saba Kurtom, Vrishketan Sethi, Ejas P. Bava, A.K. Gutierrez-Garcia, Utpreksha Vaish, Dhanisha Sulekha Suresh, Preeti Sahay, Dujon Edwards, Jumana Afghani, Satwikreddy Putluri, Karthik Reddy Kami Reddy, Chandra Sekhar Amara, Abu Hena Mustafa Kamal, Anthony Fodor, and Vikas Dudeja

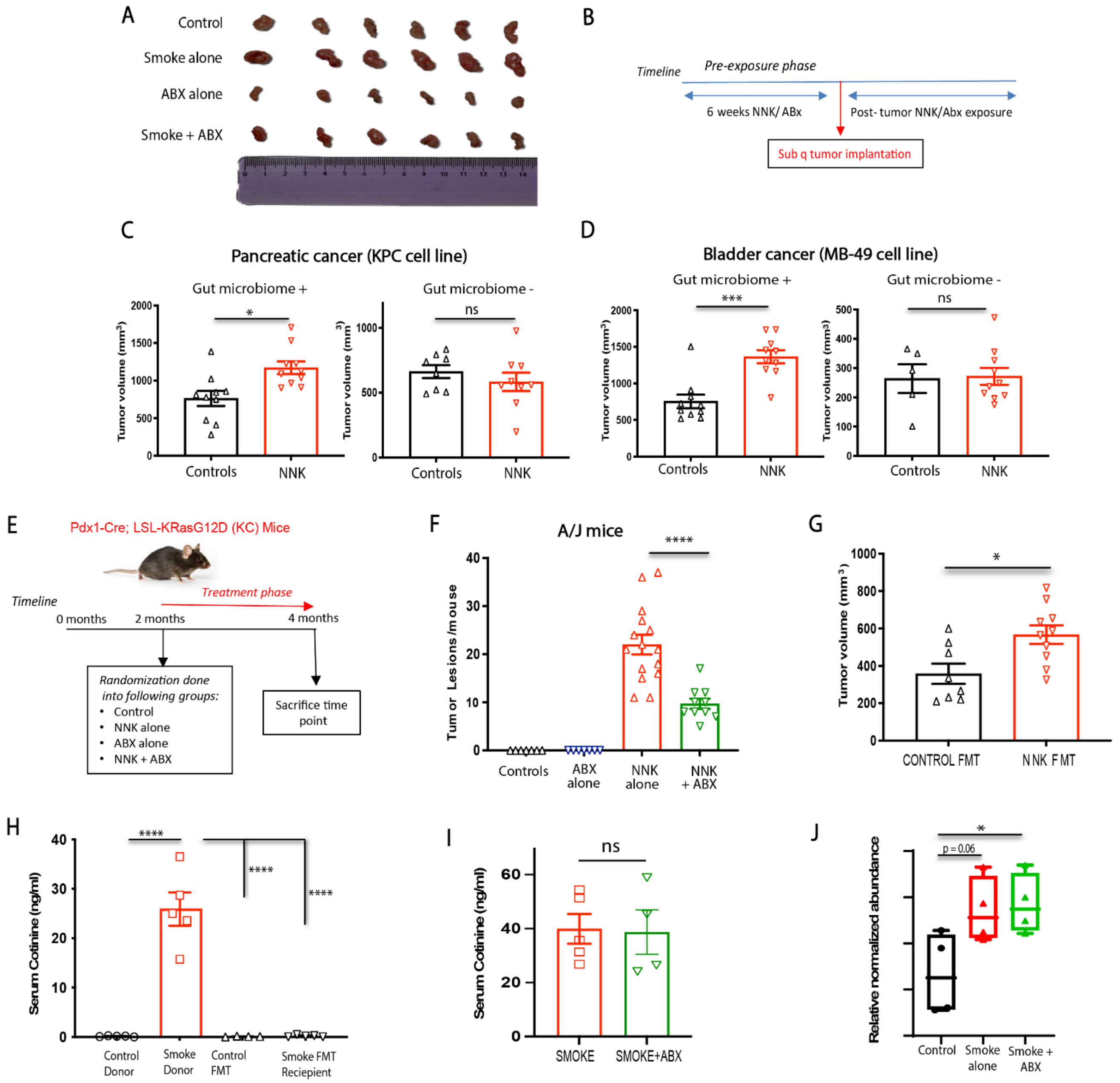

Figure S1. The gut microbiome is required and sufficient to promote cigarette smoke induced cancer progression. (Also refer to Fig 1) (A) Representative gross image of subcutaneous KPC tumors at endpoint from mice exposed to room air (control) or CSE with or without antibiotics cocktail treatment. **(B)** Schematic timeline of experimental design of the NNK exposure model in C57BL/6J mice. **(C-D)** Subcutaneous tumor volumes at the endpoint in C57BL/6J mice exposed to NNK with or without gut microbiome depletion using **(C)** pancreatic cancer and **(D)** bladder cancer (n=5-10 per group). **(E)** Schematic timeline of experimental design of the NNK exposure model in KC mice. **(F)** Individual tumor nodule counts in the lungs of A/J mice exposed to NNK with or without gut microbiome ablation with broad-spectrum antibiotics at the experimental endpoint (n = 7-15 per group). **(G)** Subcutaneous tumor volumes at the endpoint in recipient mice getting FMT from Control donors vs. NNK donors (n= 8-10 per group). **(H)** Serum cotinine concentration in mice receiving CSE or FMT from CSE donors, (n=4 per group). **(I)** Serum cotinine concentrations in mice exposed to cigarette smoke with or without antibiotic cocktail treatment (n= 4-5 per group). **(J)** Relative abundance of nicotine in ileal luminal contents in control mice (exposed to room air), CSE mice and CSE mice treated with antibiotics cocktail. CSE increased luminal nicotine content, however, antibiotics treatment did not change it further. CSE: cigarette smoke exposure; NNK: *N*-methyl-*N*-(4-oxo-4-pyridin-3-ylbutyl)nitrous amide; FMT: fecal microbiome transplant. * p-value < 0.05, *** p-value < 0.001, **** p-value < 0.0001. Unpaired t-test or ANOVA used as appropriate for statistical comparison

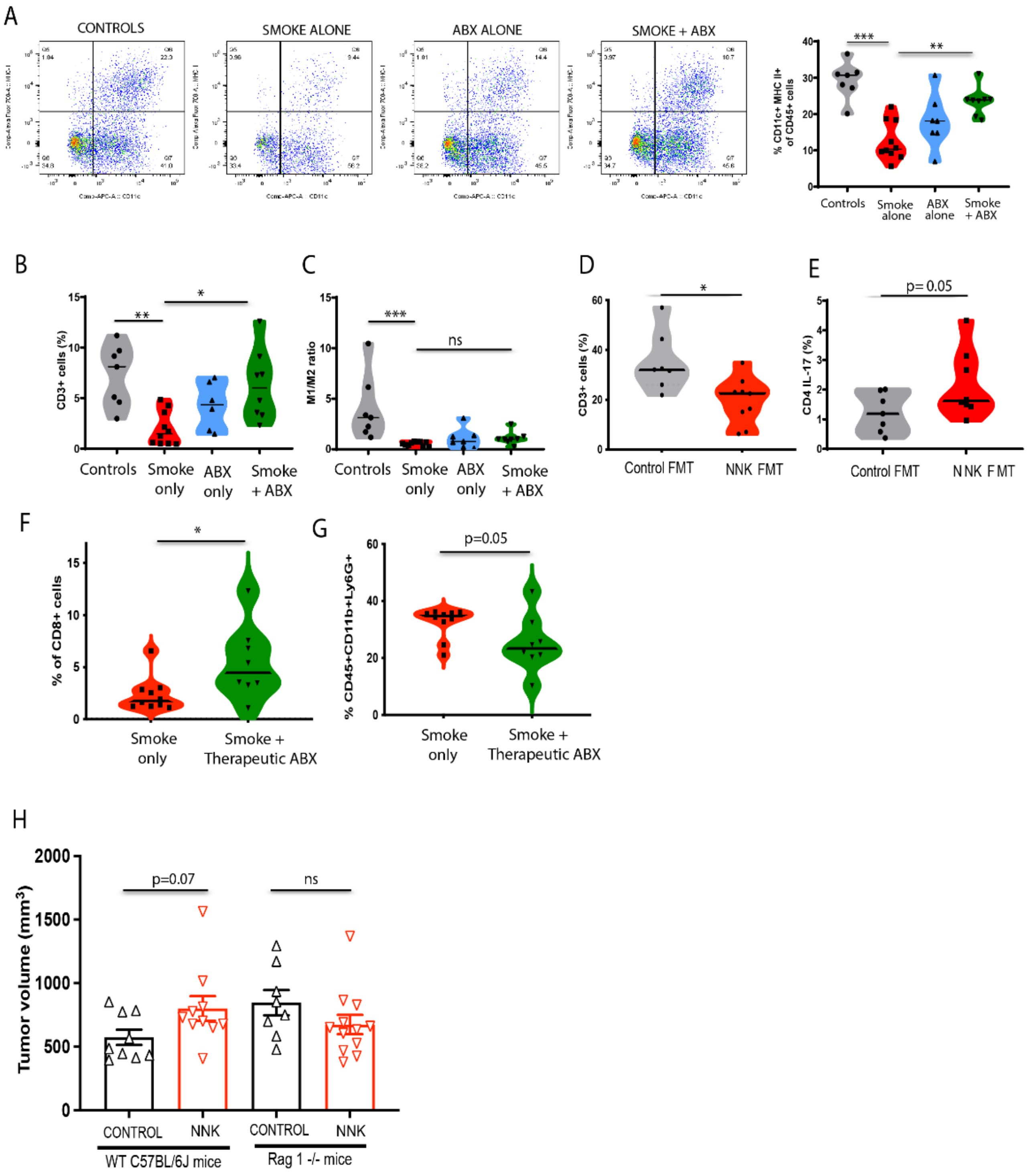

Figure S2. Cigarette smoke induced gut microbial dysbiosis is accompanied by an immunosuppressive tumor microenvironment. (Also refer to Fig 2) (A-C) Flow cytometric analysis of KPC subcutaneous tumors from control or CSE mice with or without gut microbiome depletion (n=7-9 per group). **(A)** Dendritic Cells (CD45+ CD11c+ MHCII+). Representative scatter plots and bar graph quantifications are shown. **(B)** CD45+ CD3+ cells **(C)** Ratio of M1(CD45+F4/80+MHCII+CD206-) to M2 (CD45+F4/80+MHCII-CD206+) macrophages. **(D-E)** Flow cytometric analysis of KPC subcutaneous tumors from recipient mice receiving control (n=7) or NNK FMT (n=9). **(D)** CD45+ CD3+ cells **(E)** Th17 (CD4+ IL17+) cells. **(F-G)** Flow cytometric analysis of KPC subcutaneous tumors from CSE mice (n=10) vs. CSE mice treated with therapeutic antibiotics (n=8). **(F)** CD8+ T-cells **(G)** MDSCs (CD45+ CD11b+ Ly6G+) **(H)** Subcutaneous KPC tumor volumes at the endpoint in C57BL/6J vs. Rag1-KO mice exposed to NNK, n=8-12 per group. CSE: cigarette smoke exposure; NNK: *N*-methyl-*N*-(4-oxo-4-pyridin-3-ylbutyl)nitrous amide; FMT: fecal microbiome transplant. * p-value < 0.05, ** p-value < 0.01, *** p-value < 0.001, ns=non-significant. Unpaired t-test or ANOVA used as appropriate for statistical comparisons

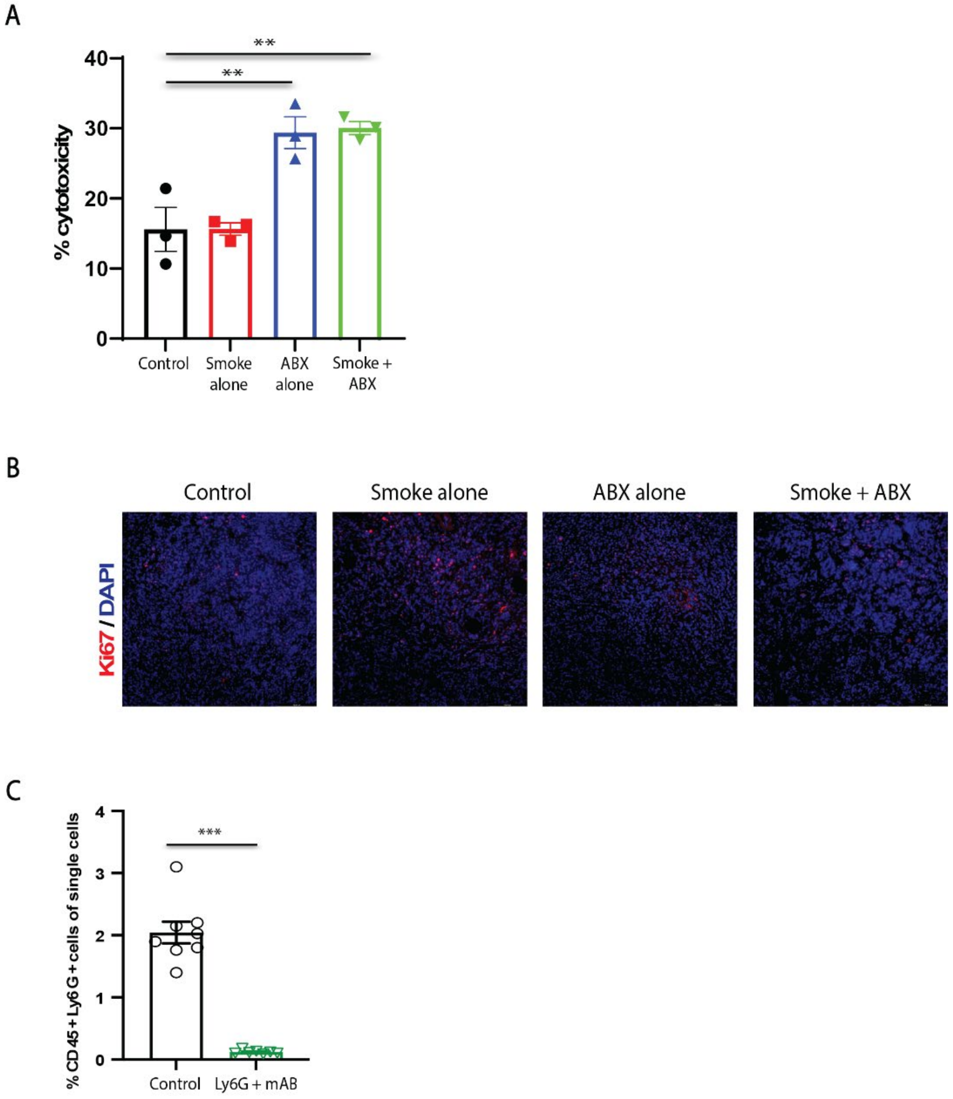

Figure S3. Smoking induced gut microbial dysbiosis affects both adaptive and innate immune response. (Also refer to Fig 2) **(A)** Splenic CD8+ T-cells were isolated using MACS sorting from tumor bearing mice exposed to room air (control) or CSE (smoke alone) with or without antibiotics cocktail at experimental endpoint. CD8+ T-cells were incubated with Calcein labelled KPC cancer cells *ex-vivo* for 4hrs and subsequently, calcein fluorescence was measured at ex 485nm/em 530nm. Percentage cytotoxicity (see methods for calculation) is plotted. n=3 per group. (**B**) Ki67 (red) staining of sections obtained from subcutaneous KPC tumors. **(C)** At experimental endpoint, flow cytometry was performed on spleens from mice treated with anti-Ly6G mAb or isotype control to confirm depletion of Ly6G+ myeloid cells *in-vivo*. ** $p < 0.01$, *** $p < 0.001$, ns = not significant. Unpaired t-test or ANOVA used as appropriate for statistical comparison

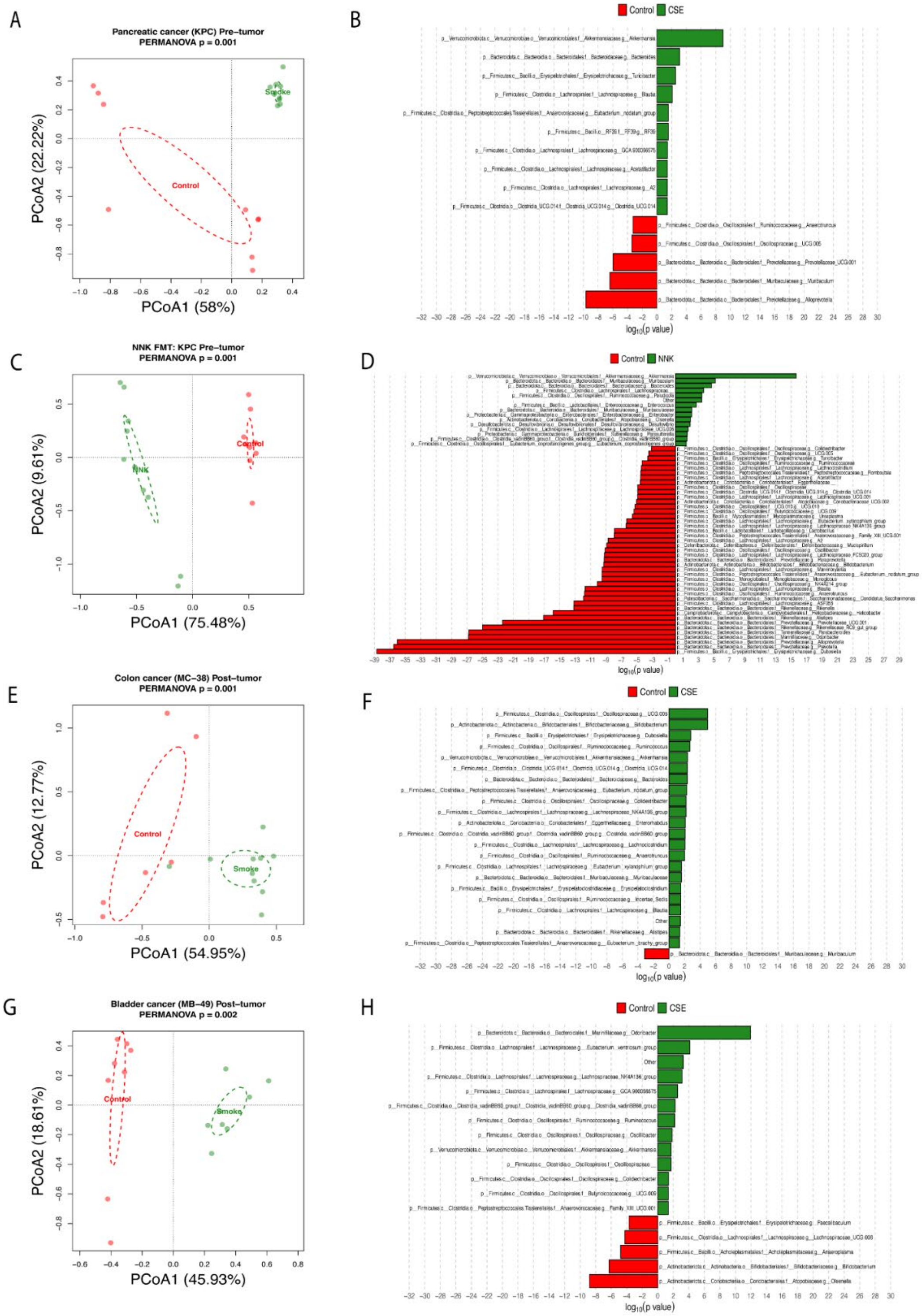

Figure S4. Cigarette smoke exposure leads to a unique gut microbial signature. (Also refer to Fig 3) (A-B) Fecal samples obtained from tumor-naive control (n=9) and CSE (n=8) mice were analyzed using 16s rRNA amplicon sequencing. **(A)** PCoA plot comparing beta diversity between control (red) and CSE (green) groups. Distance calculated using Bray-Curtis analysis, PERMANOVA used for statistical significance **(B)** Linear model analysis showing differentially enriched microbes. **(C-D)** 16s rRNA analysis of fecal samples from tumor-naive control (n=8) and NNK exposed (n=10) mice, PCoA plot **(C)** and linear model analysis **(D)**. **(E-F)** PCoA **(E)** and linear model **(F)** analysis of fecal samples obtained from subcutaneous colon cancer-bearing control (n=8) and CSE (n=11) mice. **(G-H)** PCoA **(G)** and linear model **(H)** analysis of fecal samples obtained from subcutaneous bladder cancer bearing control (n=8) and CSE (n=8) mice. FMT: fecal microbiota transplant; PCoA: principle coordinate analysis; CSE: cigarette smoke exposure; PERMANOVA: Permutational Analysis of Variance; LeFSe: Linear discriminant analysis effect size; NNK: *N*-methyl-*N*-(4-oxo-4-pyridin-3-ylbutyl)nitrous amide

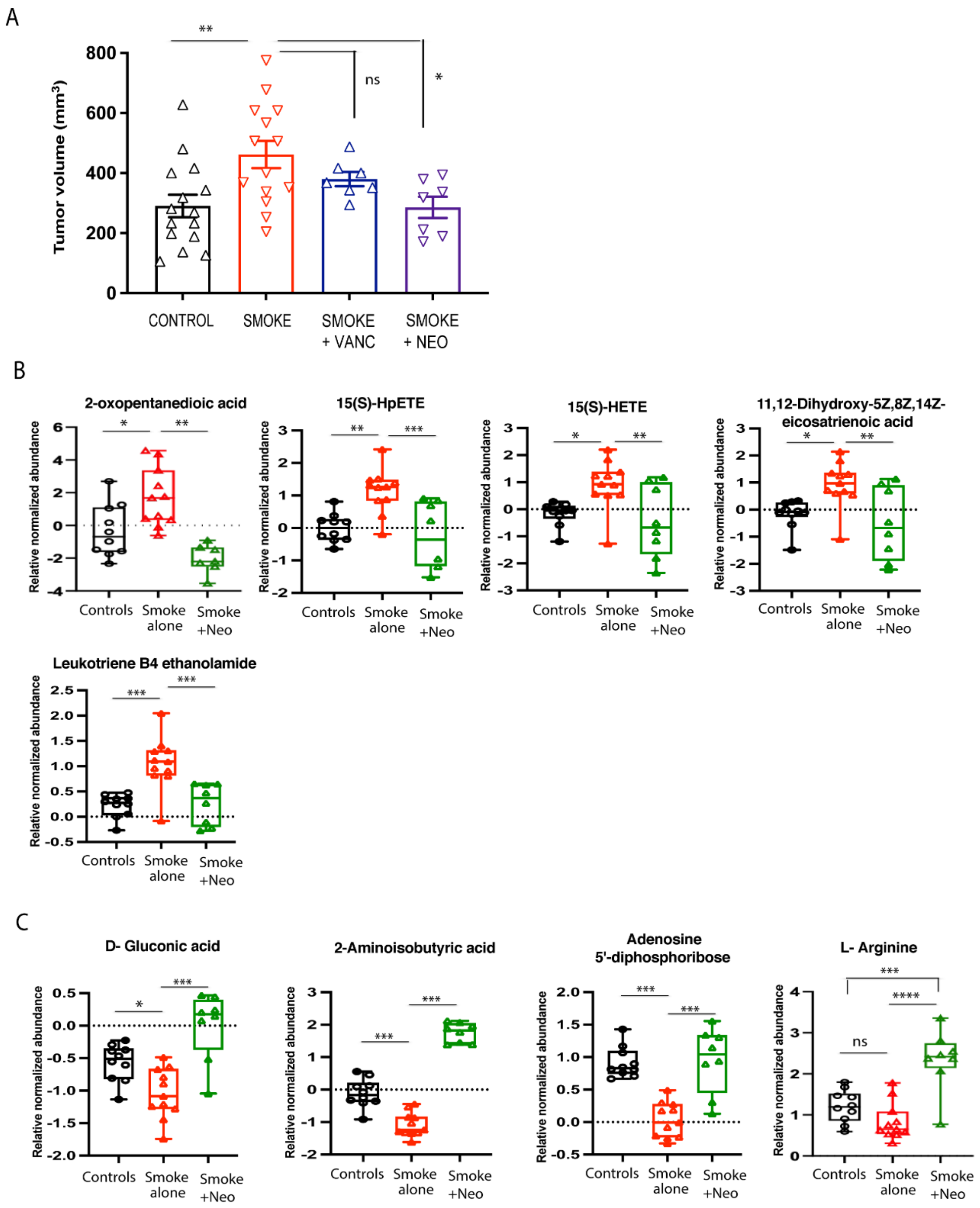

Figure S5. Cigarette smoke exposure leads to significant alterations in the gut metabolome. (Also refer to Fig 3). (A) Subcutaneous KPC tumor volumes at the endpoint in CSE mice treated with vancomycin vs. neomycin (n=7-14 per group) (**B-C)** Examples of fecal metabolites which are normalized upon treatment with neomycin (n=8-11 per group). **(B)** Metabolites that are upregulated upon CSE but are decreased in the Smoke+Neomycin group. **(C)** Metabolites that are downregulated upon CSE but are increased in the Smoke+Neomycin group. FMT: fecal microbiota transplant; PCoA: principle coordinate analysis; CSE: cigarette smoke exposure. * p-value < 0.05, ** p-value < 0.01, *** p-value < 0.001, **** p-value <0.0001, ns=non-significant. Unpaired t-test or ANOVA used as appropriate for statistical comparisons

Table S2: List of reagents

REAGENT or RESOURCE	SOURCE	IDENTIFIER
FC: anti-mouse CD45	Biolegend	Clone:30-F11
FC: anti-mouse CD3e	Biolegend	Clone:145-2C11
FC: anti-mouse CD4	Biolegend	Clone: RM4-5
FC: anti-mouse CD8	Biolegend	Clone:53-6.7
FC: anti-mouse Ly6G	Biolegend	Clone: 1A8
FC: anti-mouse CD11b	Biolegend	Clone: M1/70
FC: anti-mouse MHC II	Biolegend	Clone: M5/114.15.2
FC: anti-mouse F4/80	Biolegend	Clone: BM8
FC: anti-mouse CD25	Biolegend	Clone: PC61
FC: anti-mouse CD44	Biolegend	Clone: 1M7
FC: anti-mouse CD11c	Biolegend	Clone: N418
FC: anti-mouse TNF-a	Biolegend	Clone: MP6-XT22
FC: anti-mouse CD206	Biolegend	Clone: C068C2
FC: anti-mouse CD62L	Biolegend	Clone: MEL-14
FC: anti-mouse IL-17 A	Biolegend	Clone: TC11-18H10.1
IF: anti-mouse-Krt19	Abcam	Catalog # Ab52625
IF: anti-mouse-Ki67	Invitrogen	Catalog# MA5-14520
